# Gene Regulatory Networks Reconstruction Using the Flooding-Pruning Hill-Climbing Algorithm

**DOI:** 10.3390/genes9070342

**Published:** 2018-07-06

**Authors:** Linlin Xing, Maozu Guo, Xiaoyan Liu, Chunyu Wang, Lei Zhang

**Affiliations:** 1 School of Computer Science and Technology, Harbin Institute of Technology, Harbin 150001, China; xinglinlin@hit.edu.cn (L.X.); liuxiaoyan@hit.edu.cn (X.L.); chunyu@hit.edu.cn (C.W.); 2 School of Electrical and Information Engineering, Beijing University of Civil Engineering and Architecture, Beijing 100044, China; lei.zhang@bucea.edu.cn; 3 Beijing Key Laboratory of Intelligent Processing for Building Big Data, Beijing 100044, China

**Keywords:** gene regulatory networks, flooding-pruning hill-climbing algorithm, neighbor selection, data processing inequality

## Abstract

The explosion of genomic data provides new opportunities to improve the task of gene regulatory network reconstruction. Because of its inherent probability character, the Bayesian network is one of the most promising methods. However, excessive computation time and the requirements of a large number of biological samples reduce its effectiveness and application to gene regulatory network reconstruction. In this paper, Flooding-Pruning Hill-Climbing algorithm (FPHC) is proposed as a novel hybrid method based on Bayesian networks for gene regulatory networks reconstruction. On the basis of our previous work, we propose the concept of DPI Level based on data processing inequality (DPI) to better identify neighbors of each gene on the lack of enough biological samples. Then, we use the search-and-score approach to learn the final network structure in the restricted search space. We first analyze and validate the effectiveness of FPHC in theory. Then, extensive comparison experiments are carried out on known Bayesian networks and biological networks from the DREAM (Dialogue on Reverse Engineering Assessment and Methods) challenge. The results show that the FPHC algorithm, under recommended parameters, outperforms, on average, the original hill climbing and Max-Min Hill-Climbing (MMHC) methods with respect to the network structure and running time. In addition, our results show that FPHC is more suitable for gene regulatory network reconstruction with limited data.

## 1. Introduction

The growth and development of organisms and the ability to respond to environmental conditions are controlled by an intrinsic regulation mechanism, which spans multiple molecular levels [1]. Gene regulatory networks (GRNs) depict this complex mechanism at the level of genes and provide an intuitive understanding of how these interactions determine the characteristics of organisms [2]. The structure of GRN reflects the interactions between the regulatory elements in biological systems, such as genes and proteins [3,4,5]. Therefore, the reconstruction of gene regulatory network from gene expression data, also known as reverse engineering, is the most fascinating task in system biology and bioinformatics. More importantly, these predicted networks can generate valuable hypotheses to promote further biological research. This has led to the fast development of computational approaches for the reconstruction of GRNs [6].

At the simplest level, clustering methods can be applied to find genes sharing the same expression pattern, which are likely to be involved in the same regulatory processes [7]. Pearson correlation coefficient and mutual information [8,9,10] are commonly used metrics for measuring the similarity of expression profiles. This idea was developed further to construct relevance networks by using such metrics [11,12,13]. These correlation-based methods are highly efficient, but cannot identify the directions or model system dynamics.

The explosion of expression data, promoted by novel and high-throughput technologies, has thus propelled the evolution of GRN analysis from clustering method to systematic or model-based methods. Systematic methods can provide researchers with deeper insights into the holistic regulatory mechanism at a network level [4,14,15]. Taking this one step further, biologists can put forward valuable clues or ideas in real life scenarios, such as disease gene discovery [5], seed oil [16], yield character location [17].

However, due to the small sample size of expression data and the exponential solution space, it would be a big challenge. The large amount of data from high-throughput technologies offers an opportunity for seeking systematic approaches to understanding the structure of gene regulatory networks [4]. The main mathematical models include Boolean network [18,19,20], Bayesian Network [21,22,23,24], and differential equation [25,26,27]. These mathematical models can vary from the very simplified, such as Boolean network, to the very complex, such as differential equations in computing complexity aspect. Each has its own characteristics and fits different applicable scenes. With the gradual increase of computing complexity, the data size they can process rapidly goes down. Using the assumption that genes are simple binary switches in genetic regulation, the Boolean network approach uses the Boolean functions to model gene regulatory networks. The probabilistic Boolean network extends Boolean network methods by integrating rule-based dependencies between variables [28]. Although these assumptions make the Boolean network the most mathematically tractable, these crude simplifications cannot reflect the genetic reality. At the opposite end, the differential equation approach quantitatively describes the dynamics of change of each gene’s expression level on its regulatory genes. Obviously, differential equation approach can capture more detail about genes’ activities, but it is only suited for some concrete and very small systems, generally ascribed to computational complexity.

Bayesian network is another popular model for the reconstruction of GRNs, which uses Directed Acyclic Graph (DAG) to represent GRNs. Between these three approaches, the Bayesian network is a combination of probability and graph theory and of medium complexity and scale. More importantly, due to its inherent probabilistic nature, the Bayesian network can deal with noisy data and cope with uncertainty. Yet, the Bayesian network model has its limitations. Due to its NP-hard nature of structure learning with respect to the number of genes, the exact Bayesian network structure can be learned only for relatively small networks [29].

Typically, two main approaches, search-and-score approach and constraint-based approach, are used to learn the structure of BN. The constraint-based approach [30,31,32,33] tries to use statistical or information measures to test the conditional independence (CI) between variables. These methods rely heavily on the threshold selected for CI tests. High-order CI tests using large condition sets may be unreliable with the limitation of data size. Recently, the recursive autonomy identification (RAI) algorithm [31] reduces the number of high-order CI tests using sequentialization and recursion techniques and, hence, requires fewer data. However, the requirements of computational capabilities and data size to perform reliable tests are still problems of constraint-based approaches, which limits their application in biological network reconstruction.

The search-and-score approach [34,35,36] attempts to traverse all possible structures using certain search algorithms to find the optimal one that maximizes the scoring function. In addition, prior knowledge can be easily incorporated into the model through the prior probability term in the scoring function. The search methods are heuristic and do not guarantee global optimal in most cases in consideration of large search space.

Hence, a typical way to accelerate the learning phase of search-and-score algorithms is to reduce the search space of possible structures. Specific methods include Sparse candidate (SC) [21], maximum number of parents limitation [37] (also called maxP technique), Max-Min Hill-Climbing (MMHC) [38], and the Candidate auto selection method (CAS) [39] etc.

The sparse candidate algorithm applies a restrict-search iteration strategy to speed up the search process. It heuristically estimates the parents of each node, and then perform a search-and-score procedure. The algorithm constraints the size of the parent set per node to a user-defined upper limit *k* and repeatedly execute the above two steps. The maxP technique restricts the search space in a more simple way, by only limiting the maximum number of parents for each node without a heuristic estimation step. Nevertheless, the two methods ignore the biological fact and suffer from the problem of tuning parameter. The fact that they actually do not generate candidate sets for each node results in wasting much of the time on examining unreasonable candidates due to the scale and the sparsity of biological networks [39].

Actually, the MMHC algorithm should be called a hybrid method. It first applies a constraint-based algorithm, called Max-Min Parents and Children (MMPC), to learn the neighbors of each node and then uses hill-climbing search to orient the edges [38]. The combination of constraint-based approach and search-and-score approach seems to be a trend. So far, MMHC outperforms all other methods in the field of search-and-score approach, given enough samples.

However, as mentioned in the analysis of constraint-based approach, the exponential data dependence to accurately estimate the strength of conditional independence in MMPC step cannot be satisfied in a biological sense. The lack of data leads to a failure in candidate sets identification and then makes the behavior of MMHC algorithm similar to typical greedy search. So, we can see that the situation, typically known as “large *p*, small *n*” problem, greatly limits the use of the MMHC method for biological network reconstruction. Moreover, users also need to define a *p*-value as the threshold of conditional independence. Hence, the MMHC algorithm does not work well on small datasets and may lead to a huge amount of false positives.

Recently, in our previous work, the CAS algorithm was proposed to infer GRN [39]. It uses a method based on mutual information and breakpoint detection to separate related nodes and unrelated nodes. However, the overestimation of the strength between genes and its neighbors results in the problem of false positives.

In this paper, we propose the Flooding-pruning Hill-Climbing algorithm (FPHC) on the basis of our previous work in order to further improve its performance for GRN reconstruction. FPHC also follows the idea of hybrid methods for learning structure of BN and aims at reducing the false discovery rate of neighbor node identification. FPHC tries to alleviate these problems listed above with less data, that is, to infer the directed network with less false-positive edges and with high computational efficiency. By learning the skeleton of BN, FPHC estimates the candidate neighbor set of each node: a neighbor set of node X is all the nodes Y that shares an edge with X. The difference from existing methods is that the algorithm needs smaller sample size and is highly effective and deterministic.

FPHC works in a sound manner and solves the problems in SC and MMHC methods. To find the candidate set, the FPHC method employs a two-phase algorithm to identify the neighbors of each node, called the flooding phase and pruning phase. This is very different with MMHC, which uses a heuristic constraint-based algorithm to identify the skeleton. The flooding phase aims at finding the propagation boundary of mutual information in a DAG through a breakpoint detection method, which is the same as our previous work. In this step, the related nodes of each node will be selected. In the pruning phase, the concept of data processing level (DPI Level) is introduced to filter out indirect regulations (false positives). To infer the structure, we apply the greedy search-and-score methods to learn the network structure based on the neighbor sets of each node.

To evaluate the proposed methods, comparison experiments against several typical methods are carried out on various networks and sample sizes. Our study also provides a theoretical analysis and comparison of existing candidate selection algorithms.

## 2. Materials and Methods

A Bayesian network consists of two parts. A DAG G=(V,E) represents the network structure, where $V=\{X_{1},X_{2},\ldots ,X_{n}\}$ represents the variables (or genes), n is the total number of variables or genes and E, the edge set, consists of ordered pairs (for example, $<X_{i},X_{j}>$) of distinct nodes in V. An uppercase letter (for example, X) and $X=\left\{x_{1},x_{2},\ldots ,x_{n}\right\}$ represents a variable or gene and the corresponding expression vector. A Conditional Probability Table (CPT) represents the corresponding joint probability of this structure. Under the decomposable assumption, the joint probability can be represented in a product form:

(1) $$P(X_{1},X_{2},\ldots ,X_{n})=\prod_{i=1}^{n}P(X_{i},\mathrm{Pa}(X_{i}))$$

Then some scoring functions are derived for learning Bayesian network structures in a search-and-score scheme. In our study, the widely used BDeu score [36] (Bayesian Dirichlet with uniform priors) with an equivalent sample size of one is selected. The learning aim is to recover the parents $\mathrm{Pa}(X_{i})$ of all nodes that maximize the joint probability, given dataset *D*.

### 2.1. Mutual Information

Mutual information (MI) is a powerful and fair metric for measuring the non-linear dependency between gene pairs. In this article, mutual information is adopted as a similarity measure between the expression profiles of two genes. The mutual information of two discrete variables (genes) X,Y is defined as:

(2) $$\mathrm{MI}(X;Y)=\sum_{x\in X}\sum_{y\in Y}p(x,y)log\left(\frac{p(x,y)}{p(x)p(y)}\right)$$

where p(x,y) is the joint probability of variable *X* and *Y*, and p(x) and p(y) are the marginal probabilities of variable *X* and *Y*, respectively. The MI indicates the relationship between two genes without considering other variables. Hence, MI tends to overestimate the regulation strengths between genes. High MI value indicates that there may be a close relationship between the variables (genes) *X* and *Y*, while low MI value implies their independence. We use $\phi_{i,j}$ to represent the interaction of gene *i* and gene *j*, if $\phi_{i,j}=0$, gene *i* and gene *j* do not interact directly. This notation includes the gene pairs that are statistically independent (MI = 0) and the gene pairs that are not statistically independent and indirectly interact.

### 2.2. Data Processing Inequality and the Concept of DPI Level

The data processing inequality (DPI) states that if gene $X_{i}$ and gene $X_{k}$ interact only through a third gene $X_{j}$, and no alternative path exists between gene $X_{i}$ and gene $X_{k}$, then the following conclusion will be obtained [40,41]:

(3) $$\mathrm{MI}(X_{i};X_{k})\leq min(\mathrm{MI}(X_{i};X_{j}),\mathrm{MI}(X_{j};X_{k}))$$

That is, only the least of the three MIs can come from indirect interactions, and check the DPI may identify those indirect interacted gene pairs. Taking this one step further, we put forward the idea of DPI Level. Given target node $X_{i}$, suppose there are *l* nodes on the path from $X_{i}$ to $X_{k}$. Hence, for node $X_{k}$, this inequation can be tested *l* times. This concept uses the number of times that $X_{k}$ satisfied the inequation to indicate the distance between $X_{i}$ and $X_{k}$. Thus, the bigger the DPI Level, the more distantly related of the two nodes. Specifically, in a tree structure, the DPI Level is the jumps from $X_{i}$ to $X_{k}$, and all the nodes with DPI Level = 1 are the neighbors. 

**Figure 1:**
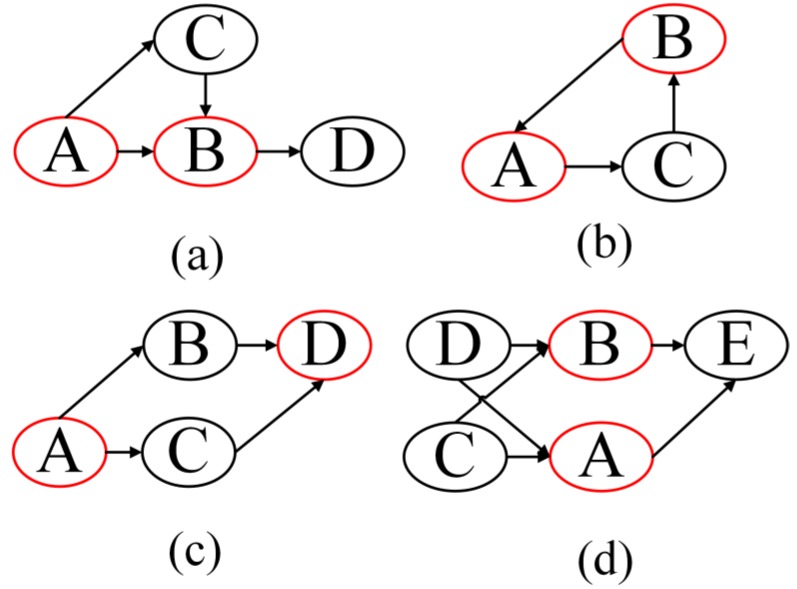
The four kinds of duplicate paths.

However, feed-forward control and feedback loops, called triples, are commonly observed in a common biological network. In Figure 1, the nodes with red cycles around it are the pairs affected by duplicate paths. They are feed forward (Figure 1a), feedback (Figure 1b), parallel control channel (Figure 1c), and duplicate parents (Figure 1d), respectively. In this situation, the computation of DPI Level is not exactly the distance between $X_{i}$ and $X_{k}$. The DPI Level of $X_{k}$ will be affected if there are triples or parallel regulations in the path from $X_{i}$ to $X_{k}$, as shown in Figure 1. The DPI Level will increase, according to the nodes involved in triples. The detailed algorithm for calculating the DPI Level is the Pruning Phase section. 

### 2.3. The Flooding-Pruning Neighbor Selection Algorithm

The Bayesian network learning algorithm FPHC presented in this work is based on the local neighbor selection algorithm called Flooding-Pruning Neighbor Selection (FPNS). FPNS provided a way to exactly identify the neighbors of target node T, that is, the edges to and from node T, and is used by FPHC to select the neighbors of each node. Although we use the break-point detection algorithm of our previous work, the idea of FPNS is different with CAS. The idea of FPNS algorithm comes from the information spreading process on a DAG. The FPNS simulates the propagation, truncation, and attenuation processes of signals. This makes the two phases of FPNS tightly coupled. FPNS consists of two phases: the flooding phase and pruning phase. The name “flooding phase” means that we will include all the nodes that can be reached when signals travel along the active paths in a flooding manner. The name “pruning phase” means that we will prune the rambling branches to remain a more precise candidate set for each target node. 

We describe the idea of FPNS briefly and then state the specific algorithm. 

For selected target node T, we represent the set of candidate neighbors of T as Nt and the set of related nodes as Rt. Given dataset D, FPNS will output the candidate neighbor set Nt, provided there is an underlying true network. Nt includes the parent nodes and child nodes of target node T. Firstly, FPNS will identify the propagation boundary of target node ***T*** in the flooding phase, that is, to generate related node set Rt. All the statistically related nodes will be included in Rt. Then, candidate neighbor set Nt is generated by cutting off the indirect nodes based on the concept of DPI Level in pruning phase. This simulates the attenuation process of signals. By invoking FPNS on all nodes, we will obtain the reduced search space, which consists of neighbor sets of all nodes. Finally, one has to get the orientation of each edge to learn the structure of BN. This will be discussed in the structure learning section.

#### 2.3.1. Flooding Phase

**Figure 2:**
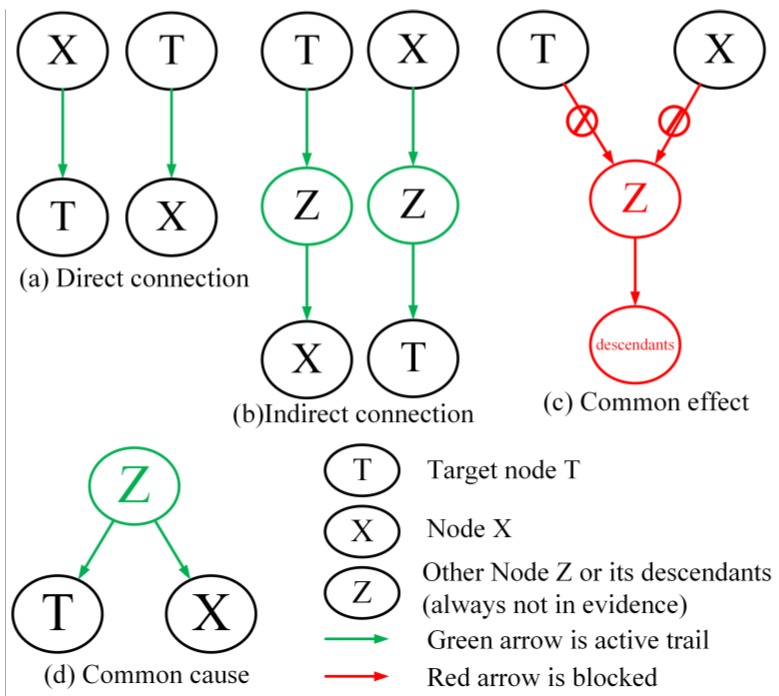
The relationships between target node T and other nodes.

We know that distant relatives with lower MI give little information for target variable T, and close relatives (neighbors) with higher MI can help to understand the target T. The aim of this phase is to identify the related nodes Rt of target node T. All the possible relations between target node T and other nodes are shown in Figure 2.

For the task of BN structure reconstruction, Node Z and its descendants are always not in evidence. Therefore, the related nodes that can be reached to or from target node T are in two categories: (i) direct connection and indirect connection situation as shown in Figure 2a,b, in which these nodes are its descendants and ancestors; (ii) common cause situation as shown in Figure 2d, in which these nodes are its brothers. As for the first type, the information from target node T can flow from its ancestors or to its descendants, furthermore, this information decreases with their genetic relationship. As for type ii, target T and its brothers share some information inherited from their parents.

The nodes that cannot be reached from Target node T are the nodes separated by a V-Structure or the nodes are not in the same connected-component, as shown in Figure 2c. These nodes are independent to target node T. That means the information is truncated from these nodes. Thus the target node T and unrelated nodes are statistically independent, which means that these MIs are zero. However, they are actually measurement noises. From what has been discussed above, we can safely draw the conclusion that the MIs of target node T and the related nodes are distributed differently with the MIs of target node T and separated nodes. So, based on the analysis and mentioned research [9,42], we can further assume that there is a breakpoint in the MIs of target T that can be used to distinguish the related nodes and separated nodes.

At this point, the problem of identifying the related node set Rt of target T (contains all the node in the propagation boundary) is translated into a breakpoint detection problem, which can be solved by hypothesis testing. At this point, the main idea of the flooding phase is the same as with our previous work. The idea is described as follows on target node T.

The MI vector of target node T and all the other nodes $M=\left\{x_{1},x_{2},\ldots ,x_{m}\right\},m=n-1$ is given and all the MIs are sorted into ascending order. According to the above analysis, the null hypothesis and the alternative hypothesis are stated as follows:

H0: Null hypothesis—no breakpoint exists
H1: Alternative hypothesis—one significant breakpoint exists

That is, there is a position in vector M that divided the nodes into two parts: related nodes and others.

Under the null hypothesis, if all the MIs are from the same distribution, then the probability is $\log\left(p\left(M_{1:m}|\delta \right)\right)$. Under the alternative hypothesis, there is a breakpoint at position *k* k∈[1,m] in M of target node T. Thus, the two types of nodes come from different distributions. The maximum likelihood can be defined as in Equation (4).

(4) $$ML\left(k\right)=\log\left(p\left(M_{1:k}|\delta_{1}\right)\right)+log(p(M_{k+1:m}|\delta_{2}))$$

where $p(M_{1:k}|\delta_{1}),p(M_{k+1:m}|\delta_{2})$ in Equation (4) are the probability density function, and $\delta_{1},\delta_{2}$ are the corresponding parameter.

To detect if there is a breakpoint in M, the testing statistic *Q* can be constructed as (5):

(5) $$Q=2\left[ML\left(k\right)-\log\left(p\left(M_{1:m}|\delta \right)\right)\right]$$

For calculating these probabilities, we should make reasonable assumptions about their probability distributions. As mentioned above, the MIs of target node T and unrelated nodes are actually noises. Therefore, a normal distribution is reasonable to model these MIs. We have learned from experience, however, that a normal distribution is a good choice to model unknown distributions. With this in mind, all the mentioned possibilities can be calculated correctly.

Usually, a constant *c* should be selected as a threshold to complete the hypothesis testing. Actually, to achieve the goal of identifying the best candidate of related nodes, we just need to locate the position *k* in relation to maximum *Q*. At this point, it becomes to an optimization problem, as in Equation (6):

(6) k=argmax(Q)

The nodes on the right side of position *k* are added into Rt. The nodes in set V are processed one by one.

**Theorem** **1.** If MIs can be estimated with no errors, the Flooding Phase can identify the related nodes exactly.

**Proof of Theorem** **1.** If the MIs are estimated with no errors, the MIs of target node T and the statistically dependent nodes (the nodes separated by a V-structure and the nodes in the disconnected part) will be zero. One can just add all the nodes with non-zeros MI to the Rt set. ☐

In reality, especially in biological scenes, MIs cannot be estimated exactly due to the noise and the limitation of data. According to the experiment, the Flooding phase works in practice.

#### 2.3.2. Pruning Phase

So far, we have generated the related node set Rt, which contains neighbors and indirect nodes (false positives). That is to say, we get a high Type I Error and a low Type II Error. Hence, we designed a pruning step to reduce the Type I error by removing the indirect nodes.

Recent studies [11,40] indicate that data processing inequality can help to distinguish indirect regulation relationships. However, it has some potential limitations. Based on this concept, we put forward the concept of DPI Level to rank all the related nodes of Target T that are identified in the flooding phase. The pruning phase using DPI Level to prune the false positives of target node T. The definition of DPI Level for related nodes of target T is shown as follows:

**Definition** **1.** *Given target node T and its related node set*Rt, *in which the nodes are sorted according to MI, the DPI Level of node*$X_{i}\in Rt$*is defined as follows:*

1. If $X_{i}$ is the first node in Rt, the DPI Level of $X_{i}$ is defined as 1.
2. If $X_{i}$ is not the first node in Rt, for each node $X_{a}$ in front of $X_{i}$, triplet $<T,X_{a},X_{i}>$ is constructed and tested using inequation (3). The DPI Level of $X_{i}$ is defined as the maximum DPI Level of $X_{a}$ that satisfies inequation (3) plus 1.

The DPI Level reflects the affinity between the related nodes and target node T.

According to the concept of DPI Level, we designed a pruning phase to prune the related node set Rt and then generate the neighbor set Nt.

For target node T, the pruning phase begins from the identified related nodes set Rt which consists of ordered related nodes. Starting from the second node in Rt, the pruning phase calculates the DPI Level of each related node in turn until we have traversed the set Rt. Then, we apply this procedure to all nodes in V to get the hierarchical structure of each node. 

By definition, the smaller the DPI Level, the closer the relationship is between target node T and node $X_{i}$. Obviously, the threshold of DPI Level greatly affects the result of pruning phase. Hence, the key problem is how to select the threshold of DPI Level to determine the nodes, which are the direct neighbors.

**Theorem** **2.** *Given target node T and its related node set*Rt, *if the subnetwork defined by*$G^{\prime }=(V^{\prime },E^{\prime }),V^{\prime }=\{T\}\cup Rt$*is a tree, the pruning algorithm will construct the underlying structure correctly, provided there is enough data to estimate MI correctly*.

**Proof.**  According to Theorem 1 in ARACNE [11], the underlying undirected network can be reconstructed exactly, provided the network is a tree and has only pairwise interactions. At this point, if there is no feed forward control and feedback control in this subnetwork, the underlying subnetwork defined by $G^{\prime }=(V^{\prime },E^{\prime }),V^{\prime }=\{T\}\cup R_{t}$ is a tree. Hence, we will reconstruct the structure of this subnet. ☐

Based on Theorem 2, we derive lemma 1 for the threshold of DPI Level.

**Lemma** **1** *If target node T is not involved in any feed forward or feedback loops, the DPI Level of the directed neighbors of target node T is 1*.

**Proof** **.** If target node T is not involved in any feed forward or feedback loops, there are no duplicate paths from the neighbors to target node T. That is, no triplet $<T,X_{a},X_{i}>$ satisfies Equation (3). Hence, the DPI Level of neighbors is 1. ☐

Theorem 1 and Lemma 1 give a solution under ideal circumstances. However, gene regulatory networks are not restricted to tree structures and feed forward and feedback control schemas are often observed in GRNs. At this point, applying inequation (3) will break the triples of feedback or feedforward loops. The DPI Level of neighbors will change with the number of nodes involved in loops. As mentioned above, a different threshold leads to a different depth of pruning. Moreover, the sample size and complexity of the network will affect the estimation of MI and then the calculation of DPI Levels. Details of how to select the threshold will be discussed in the next section.

So far, we have described the whole procedure of FPNS. The pseudo code of FPNS is shown in Algorithm 1. Here, we use threshold θ to denote the pruning depth in pruning phase. If the DPI Level of the node is less than this threshold, this node is selected as the neighbor node. If the DPI Level of the node is bigger than this threshold, this node is pruned.

The full algorithm is as follows.

**Algorithm 1.** The Flooding-Pruning Neighbor Selection algorithm (FPNS)**Input:** Node Set $V=\{X_{1},X_{2},\ldots ,X_{n}\}$, Discrete Expression data D, threshold θ for pruning depth **Output:** Neighbor Set of each node in V, denoted by $\left\{N_{X1},\ldots ,N_{Xn}\right\}$Initialize the neighbor set of each node with null set. **For**
i=1:n  $R_{Xi}=\emptyset$  // Flooding phase.  Candidate set $C_{i}=V/X_{i}$.  Calculate MI of $X_{i}$ and all nodes in $C_{i}$.  Store the *n* 1 MIs into ***M*** in ascending order.  //locate position *k* that maximizes statistic Q  k=argmax(Q)  **For** every node $X_{c}$ in $C_{i}$    $R_{Xi}=\{X_{c}\in C_{i}|X_{c}\;\mathrm{is}\;\mathrm{in}\;\mathrm{the}\;\mathrm{right}\;\mathrm{side}\;\mathrm{of}\;k\}$  **End For**  // Pruning phase.   Calculate the DPI Level of each node in $R_{Xi}$  For every node $X_{r}$ in $R_{Xi}$    $N_{Xi}=\{X_{r}\in R_{Xi}|\mathrm{DPI}\;\mathrm{Level}\;\mathrm{of}\;X_{r}\;\mathrm{is}\;\mathrm{less}\;\mathrm{than}\;\theta \}$  **End for** **End for** Return $\left\{N_{X1},\ldots ,N_{Xn}\right\}$


### 2.4. Choice of Tuning Parameter

The major factors that affect the selection of tuning parameter θ are the sample size and the complexity and size of the network.

(a) Sample size has a significant effect on the estimation of MI.
(b) Network size will affect the power of statistics in the flooding phase.
(c) Network complexity directly affects the calculation and selection of θ.

Moreover, we do not forget that the aim of FPNS is generating the candidate neighbor set for the following structure learning. As we know, due to the factors mentioned above, the pruning depth will affect the result of the candidate neighbors. This will directly influence the recall of true neighbors. The smaller the threshold, the more false positives are excluded. Obviously, if the DPI Level is set to one, all the false positives will be avoided. However, some true neighbors involved in feedback or feed forward loops will also be excluded from the true neighbor set.

According to the above analysis, we give some guidance on how to select θ that balances Type I and Type II errors.

For the same network, having enough data means an accurate estimation of MI. Hence, if only limited data are available, θ should be appropriately increased to get a reasonable sensitivity. For the target network of high complexity, θ should be appropriately increased because feedback or feed-forward loops in a network will affect the DPI calculation of related nodes. For the target network, one could roughly estimate its complexity according to the possible function to be studied. According to our experiment results on different networks and different sample sizes, θ=3 or 4 are suitable in most cases. For larger networks with limited data, θ=5 may be more suitable. These suggested settings are drawn on the above analysis and in our experiment results.

Do we have a more general strategy for θ instead? Here, we give a general setting for θ. Set θ as the mode of the calculated DPI Levels of nodes in Rt but no less than 2. Since MI decreases rapidly as the signals travel along an active path, we find that the distant relatives on rambling branches will be ignored. Hence, spindle-shaped shrinkage will occur during the flooding phase. This makes the reconstructed structure of Rt (the distribution of the DPI Levels) like a spindle. Hence, the mode is selected in a natural way to cut this spindle. The setting “no less than 2” is a small trick to increase the sensitivity due to the complexity of real distribution and the situation of Theorem 1. The results with this setting are also included in this work.

### 2.5. Example Trace

**Figure 3:**
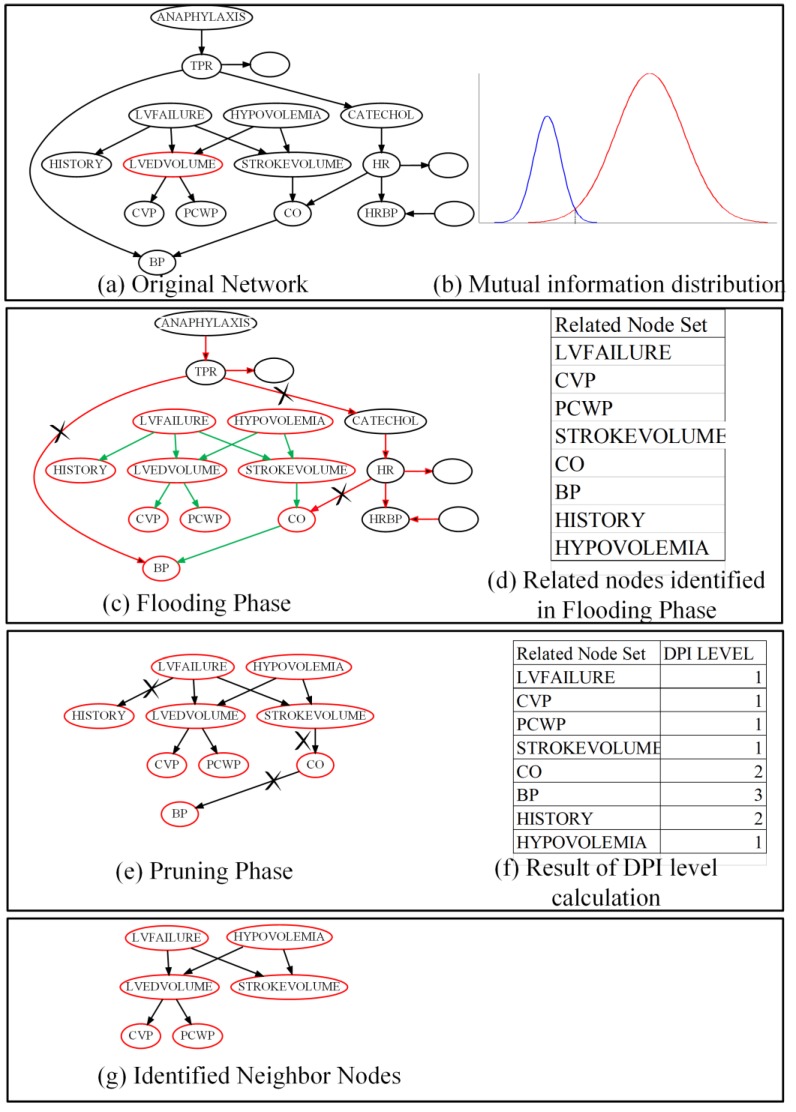
The example trace of Flooding-Pruning Hill Climbing (FPNS). DPI: data processing inequality.

We will further illustrate that FPNS runs on target node T by using an example trace shown in Figure 3. This running example is part of the alarm network with sample size = 200. In this case, we consider that the data is nearly enough for estimating the MIs correctly. The original network, part of the alarm network, is shown in Figure 3a. Target node T (LVEDVOLUME) is denoted with a red circle around it. The circles without node names indicate the rest of the alarm network. Figure 3b is a schematic diagram in order to denote that MI distribution of T and related nodes are different from the MI distribution of T and unrelated nodes. In addition, this diagram shows that we cannot decrease the Type I error and Type II error at the same time without increasing the sample size. According to the MIs distribution, we do a hypothesis test as described in the flooding phase. That is, to identify all the nodes that can be reached from target node T, as shown in Figure 2. In Figure 3c, the red arrow means blocked trails by V-structure, and the green arrow means active trails through which target node T can reach the other nodes the same as in Figure 2a,b,d. Here, nodes with a red circle around them are the identified related nodes which can be reached through the active trails. Nodes with black circles in Figure 3c means these nodes are separated by a V-structure and will be eliminated in the step. Figure 3d lists the related nodes identified in the flooding phase. Next, as shown in Figure 3e,f, FPNS will prune the related nodes according to the DPI Level of each node. Here, the pruning depth is set to 1. This means that all the nodes with a DPI Level bigger than 1 will be cut off. Finally, the identified neighbors are shown in Figure 3g. As we can see from Figure 3g, a sibling node (STROKEVOLUME) is identified as a neighbor of target node T by mistake. The correlation is over-estimated incorrectly because of the duplicate path (they share two common cause nodes) from STROKEVOLUME to target node T, as described in Figure 2 (parallel regulation). 

### 2.6. Flooding-Pruning Hill-Climbing-Structure Learning

In this section, we will illustrate FPHC for network structure learning. Firstly, the neighbors of each node are obtained by FPNS, and then we perform a greedy hill-climbing search in the restricted space. The algorithm begins with an empty graph. In each iteration, operators (add-edge, delete-edge, reverse-edge) will be tried on the graph to find the highest scoring operator. When the total score is stable, the algorithm returns the highest scoring DAG. Here, in our work, FPHC uses the BDeu score to evaluate the structure. However, FPHC performs the search in a restricted space. That means, only the edges that are included in the neighbor set will be considered. It is important to note that FPNS does not make a distinction between parents and children. Mainly because it is difficult to distinguish whether neighbor node X is a parent or a child of target node T without knowing the other nodes of the network. The structure with the highest score will be returned until the algorithm reaches convergence.

Previous studies [21,38] have shown that constraining the search space can improve the efficiency over the original greedy search-and-score method. FPHC is presented based on this idea, but an efficient neighbor selection algorithm (FPNS) is applied. In FPNS, only pairwise relationships need to be calculated for MI, as powerful metrics. Estimation of pairwise mutual information needs a smaller sample size than testing conditional independence in MMPC, which is an exponential to the size of conditioning set. The smaller sample sizes for accurately estimating MI, compared with MMPC, makes FPHC more suitable and efficient for most practical problems. In addition, the FPNS algorithm is deterministic rather than heuristic and will lead to reliable and stable outputs. Hence, FPHC is more efficient and robust. 

### 2.7. Time Complexity of FPNS Algorithms

According to the analysis, we know that the same procedures are applied to each node. For target node T, we will check the |V|−1 position to find the position k that maximizes statistic Q in the flooding phase. Hence, the time complexity is $O(|V{|}^{2})$. Suppose Rt includes all the other nodes in the worst case, the times for calculating the DPI Level of each node in Rt will be $N=\sum_{i=1}^{|V|-1}a_{i},ai=\sum_{j=1}^{i}j$. The time complexity in pruning phase will be $O(|V{|}^{3})$. Hence, in the worst-case scenario, time complexity is $O(|V{|}^{4})$. However, the FPNS algorithm runs faster than the estimated worst time, even if on a very small sample size.

## 3. Data and Performance Measures

### 3.1. Used Networks and Data Generation

To test the performance of the adaptability of the proposed FPHC method, we selected two types of networks in the evaluation, including known Bayesian networks from decision support systems and simulated biological networks from DREAM 3 challenge (Dialogue on Reverse Engineering Assessment and Methods). The known Bayesian networks include the alarm and Hailfinder network. For these networks, we generate datasets of different sample sizes (50 to 50,000) and 10 different datasets are sampled with the same sample size. The biological networks are subnetworks of 100 genes sampled from a real *E. coli* network and Yeast network. For these simulated biological networks, the data of 100 samples were generated following the guidance of the GNW software [43], the same as with the DREAM multifactorial challenge settings.

### 3.2. Performance Measures

Fair evaluation of the quality and speed of neighbor nodes selection structure learning is needed in order to illustrate the performance.

In the neighbor selection phase, we expect to rule out nonadjacent nodes to reduce the search space as much as possible. Hence, we treat the task as a classification problem and consider two different performance criteria: (a) Sensitivity (also known as recall) and (b) Specificity. They are defined as follows:

$$\begin{array}{l} Sensitivity\;=\;\frac{TP}{TP+FN}\\ Specificity\;=\;\frac{TN}{TN+FP} \end{array}$$

It should be noted that *TP* is the total identified true positives of all the nodes. Likewise, *TN*, *FP*, and *FN* are counted in the same way. We can see that sensitivity and specificity have a high correlation with the search space. Sensitivity, also known as recall, limits the upper bound of following structure learning. Specificity of neighbor selection phase affects the size of the search space and further affects the excitation time. Thus, higher sensitivity and specificity means a more exact search space. All the results are the average of 10 datasets of the same sample size in order to illustrate the stability. Moreover, in order to compare the execution speed of algorithms, we select runtime as the measurement.

For evaluating the quality of structure learning, we employ several metrics. The first and the basic one is the F-score. Moreover, we employ structure hamming distance (SHD) as another metric, which was reported by Ioannis Tsamardinos in Reference [38]. SHD directly calculates the difference of the learned and the gold-standard networks in terms of the partially directed acyclic graph (PDAG), including extra edges, missing edges and opposite edges. In addition, an undirected version of SHD, called USHD, is also used in our work. USHD just compares the skeleton of the learned and the gold-standard networks. At last, we employ running time as the standard metric in terms of execution speed.

In the results section, comparison results are normalized with the results of the greedy search algorithm to clearly show the performance changes across different methods and/or different sample sizes. Thus, a normalized sensitivity greater than 1 means a better performance than the original hill climbing method on the same task. Likewise, the specificity and the F-Score are in the same situation as sensitivity. Instead, the normalized SHD, USHD (aliased as SHD Ratio and USHD Ratio) and the running time lower than one means better performance.

## 4. Results and Discussion

In this section, we will analyze and compare the performance of FPHC algorithm from four different aspects. The FPNS algorithm restricts the search space and the upper bounds of structure learning. Its performance highly impacts the following structure learning. So, we first validate the effectiveness of the proposed neighbor selection method FPNS on different networks and different sample sizes. Comparison experiments of different situations with MMPC algorithm are also carried out. Secondly, we analyze the influence of different network and different sample sizes on the performance of FPHC algorithm. Thirdly, the proposed FPHC algorithm will be compared with the state-of-the-art MMHC algorithm, previously proposed CAS (Candidate Auto Selection) algorithm, and the original Hill-Climbing algorithm according to various evaluation metrics and recommended parameters. Finally, the proposed algorithm will be applied to a simulated biological dataset and compared with methods mentioned above.

### 4.1. Effectiveness of FPNS

We first show how the sample size affects the neighbor selection result on the known Bayesian network. Then, the comparison result with the MMPC under the selected parameters is shown.

**Figure 4:**
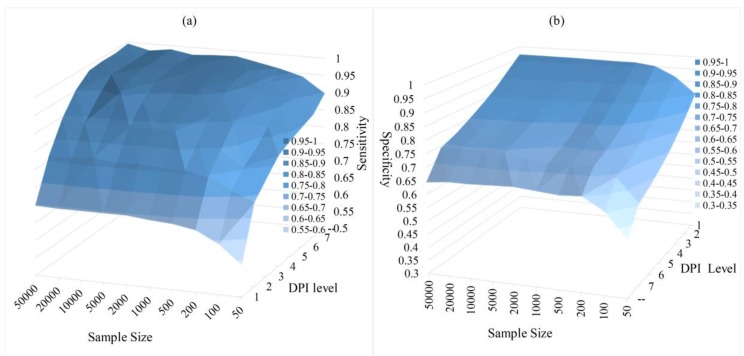
The neighbor selection results with different sample sizes and different DPI Levels. (**a**) sensitivity result in the alarm network; (**b**) specificity result in the alarm network. ‘--’ denotes no pruning.

Figure 4 shows the sensitivity and specificity of the alarm network changing with sample size and DPI Levels. In Figure 4, we use ‘--’ to denote no pruning. As we can see from Figure 4: (a) The increase in the sample size results in a continuous performance boost on each level of DPI. This is largely due to having enough data that improves the estimation of MI; (b) Given the fixed sample size, with the increase of DPI Level, the sensitivity has continued to rise and the specificity has continued to decline; (c) When we do not prune the related node set, that is, when no rambling branch is cut off, the sensitivity is the highest and the specificity is the lowest. That is, the restriction on the search space is very loose; (d) If θ is set to 1, we get a very high specificity but a lower sensitivity. This situation leads to a higher performance penalty; (e) Even if the sample size is very limited, the FPNS algorithm still shows considerable performance. These results are coincident with the analysis in the method section.

### 4.2. Comparison with Max-Min Parents and Children(MMPC)

We compare the FPNS algorithm to the MMPC algorithm, which is used in the MMHC algorithm, using the recommended parameters in the method section.

**Figure 5:**
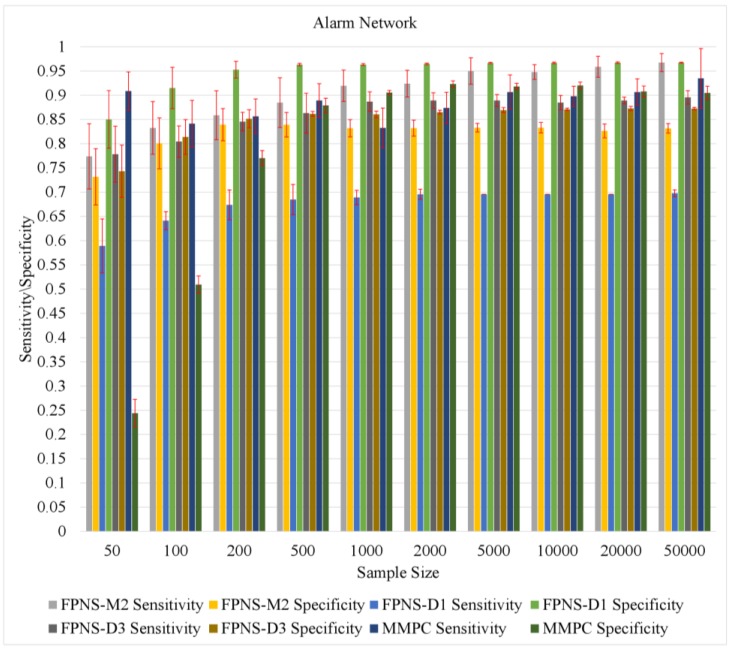
The sensitivity and specificity results in the alarm network.

In Figure 5 and the following sections, suffix ‘M2’, ‘D1’, and ‘D3’ represents different DPI Levels as parameters of the pruning phase: “’Mode bigger than 2’, ‘DPI Level 1’, ‘DPI Level 3’. These parameters are typical values, as suggested in Section 2.4. The following explanations and discussions are based on these typical values to bring about informative clues for reconstructing GRNs using FPHC.

Figure 5 shows the performance of FPNS and MMPC.

When the sample size is very limited, FPNS still shows more reasonable results than MMPC. Especially when the sample size is less than 500, MMPC selects nearly all the nodes as candidates due to the limitation of sample size as described in its original paper, as shown by the deep blue and deep green bar. When the DPI Level equals 1, the specificity is highest in all situations of different sample sizes. This result confirms the previous conclusion in the method part. As we can see from Figure 5, the typical threshold of D3 and M2 are suitable parameters, with respect to the sensitivity, as shown by the dark gray and gray bars. We can see that when the threshold is set to M2, the sensitivity keeps growing with the sample size while maintaining high specificity, as shown by gray and light yellow bars. Meanwhile, D3 gets a more stable performance with a smaller gap between the sensitivity and the specificity. When the sample size is bigger than 2000, the sensitivity of MMPC exceeds the FPNS algorithm. However, the specificity is still lower than FPNS (D1). Hence, we conclude that the performances of FPNS are better than MMPC with limited samples corresponding to sensitivity and specificity, and the performance of FPNS is still competitive in specificity.

Table 1 shows the running time of FPNS and MMPC with different sample sizes. We can see that the FPNS runs much faster than MMPC. At the same time, the runtime of FPNS does not significantly increase due to its deterministic property.

### 4.3. Comparison Results of Structure Learning

In this section, the structure learning results of MMPC, Hill Climbing (HC), and the FPHC are compared on different networks of various sample sizes. In the following section, we use HC to denote the Hill Climbing method.

#### 4.3.1. Performance Comparison on Alarm Network and Hailfinder Network

**Figure 6:**
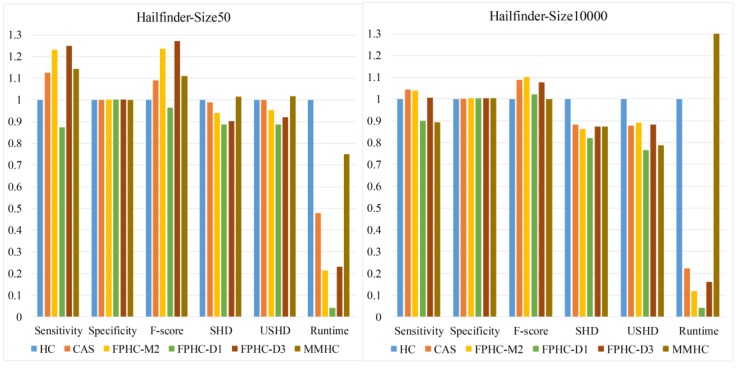
The performance comparison of different methods when sample size is 50 and 10,000.

We compare the performance of FPHC and other methods for the alarm network and Hailfinder network under different sample sizes. Figure 6 shows the comparison results with limited data and ample data from the Hailfinder networks. When the sample size is limited to 50, FPHCs with slightly bigger DPI Level (D3) obtain more true edges, and the learned network is better than MMPC. However, when the sample size is big enough, FPHCs with a slightly smaller DPI Level (M2) obtain truer edges and better performance.

**Figure 7:**
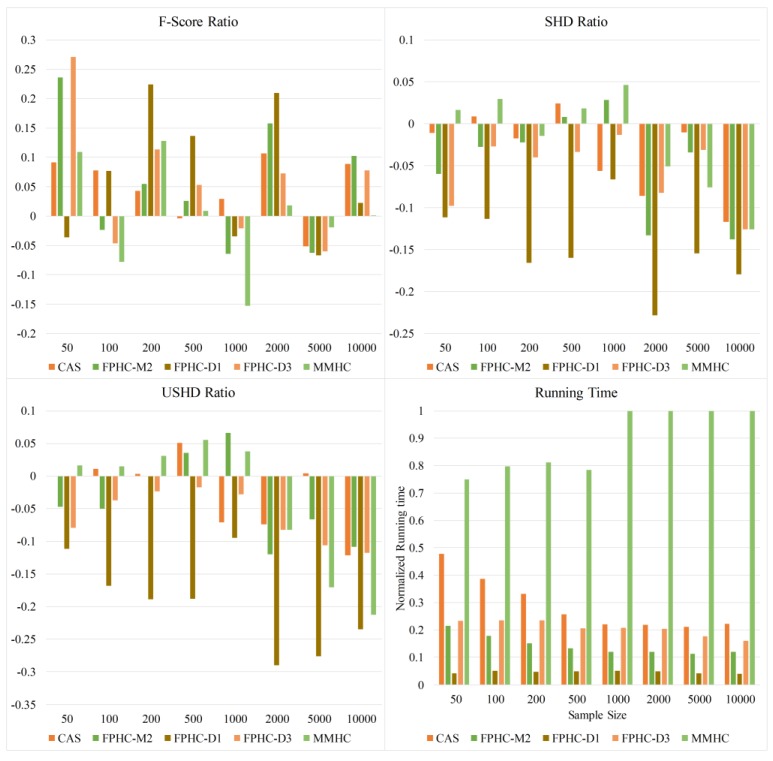
The performance comparison of different sample sizes and methods on the Hailfinder network.

Figure 7 shows the overall comparison of all sample sizes on the Hailfinder network. We can see from Figure 7 that FPHC outperforms the other methods by all the metrics. When the sample size is 50, FPHC(D3) has the highest F-Score. Then it varies with different sample size. When the sample size is less than 1000, FPHC (D1) outperforms the others according to the F-Score. With the increase of sample size, FPHC (D1) gets obvious improvements according to SHD. Obviously, FPHC (D1) get the best performance according to the SHD and USHD metrics. In addition, FPHC with the general setting M2 outperforms the MMHC method and does not appear to be much different from FPHC(D1). The growth in the number of nodes brings the improvement of statistical power. That is, it improves the performance of hypothesis testing in the flooding phase. The running time of FPHC is more stable than other methods due to the exactly reduced search space. The normalized running time gradually decreases, except for MMHC. This means the neighbor selection phase can indeed accelerate the structure learning. Nevertheless, the MMHC algorithm does not go through the same situation. We also find that when the sample size is larger than 500, the running time of MMHC is longer than the original Hill climbing. This could be because the heuristic procedure in both neighbor selection and edge orientation finds it hard to converge.

#### 4.3.2. Comparison Results of Insilco Networks

**Figure 8:**
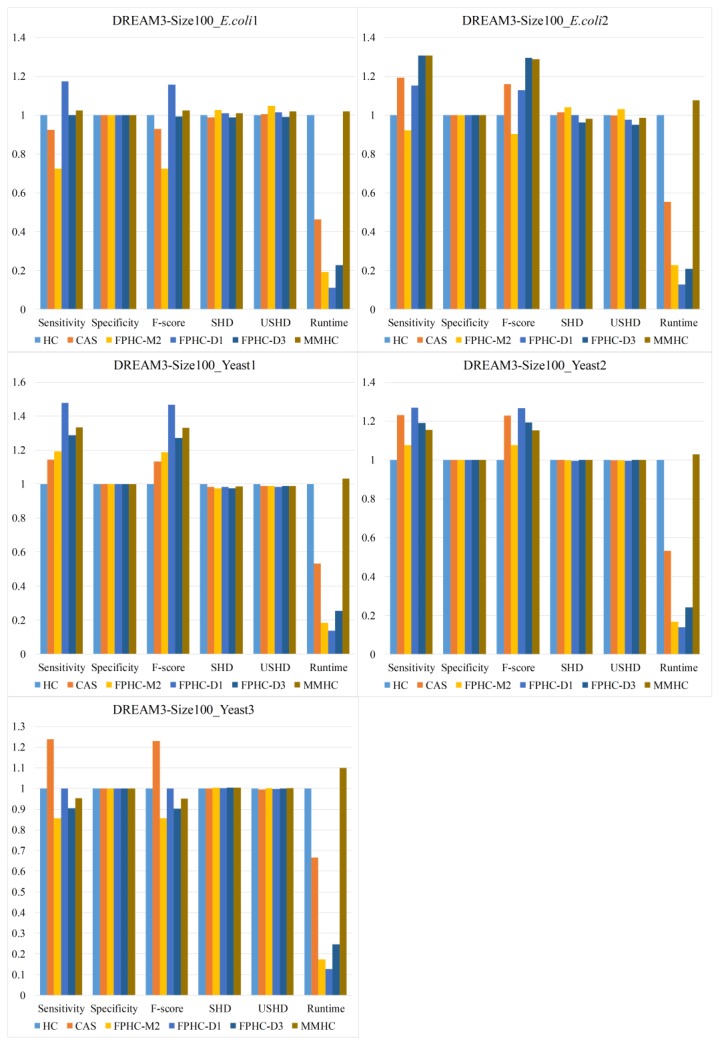
The comparison results on the DREAM networks.

Finally, we test the FPHC algorithm on simulated networks as shown in Figure 8, which are sampled from real biological networks. The three bars in the middle show the performance of FPHC with three typical parameters: D1, D3, M2. Firstly, the running time of FPHC is around one fifth, even less than that of MMPC and HC. We can find that FPHC (D1 or D3) outperforms other methods in most cases except for the yeast3 network. FPHC (M2) obtains considerable performance compared to the MMHC algorithm.

We can see from the Figure 8 that FPHC (D1) almost outperforms the other methods by the metrics of sensitivity, but it is not the best by the SHD and USHD metrics. The main reason is that the search space is unduly limited under this parameter. FPHC (D3) outperforms the other method by the metrics of SHD except for the Yeast3 network. At this point, we can look back to the suggestions in Section 2.4. For complex networks with limited sample size, the users should select a larger pruning depth.

In the Yeast3 networks, the CAS algorithm gets the best performance by the metrics of sensitivity and F-Score. However, while according to the metrics of SHD and USHD, the FPHC algorithm gets a comparable performance; the running time of FPHC is only one-third of that of the CAS algorithm. This is mainly because the Yeast3 network has 551 edges, 3–5 times more of the other networks. At this point, using DPI Level 3 as the parameter may not be suitable. However, if we rerun FPHC and set the parameter to 5, the result is nearly equal to the result of CAS and is 20 percent above the HC algorithm. In addition, more remarkably, while we have made more of a considerable progress than the original HC and MMHC algorithms, it still has a fair distance between the predicted networks and golden standard networks for complex networks such as Yeast3.

## 5. Conclusions

In this paper, we propose a novel hybrid method FPHC based on the Bayesian network for GRN reconstruction. FPHC follows the idea of the hybrid method, but uses a new neighbor selection method based on the flooding-and-pruning strategy in a workable and effective way, and then applies search-and-score approach on the reduced search space to learn the network structure. Especially, FPHC provides a new way to reduce the search space, which is more sample efficient. With this, it improves the Bayesian network learning on limited data, which is common in a biological sense and gives an opportunity to improve GRN reconstruction with less sample size. In particular, FPNS runs faster than MMPC on all the networks and datasets. In our experiments, FPHC outperforms the existing methods on small data sets and is comparable to the state-of-the-art methods with enough data. Furthermore, experiments were carried out on various biological networks. The results show that FPHC is more suitable in terms of GRN reconstruction.

As a hybrid method, FPHC uses the similar idea as described in Sparse Candidate and MMHC. However, different with common constraint-based algorithms, FPHC applies a new way to select the candidate neighbor set by using the flooding-and-pruning strategy. FPHC adopts a deterministic approach to identify the neighbors of each node and complete it in one iteration. This makes FPHS run faster than the other method. FPHC is a workable and effective algorithm on different datasets and alleviates the problems of the sparse candidate and MMPC. Experiments on various networks and datasets show that it outperforms the previously presented MMPC algorithm in the phase of neighbor selection and shows significant improvement on small datasets. In addition, the algorithm uses an adjustable parameter to satisfy different requirements. Especially as we give reasonable and detailed guidance on selecting the specified parameter.

Finally, the FPHC algorithm is workable and effective for learning a network structure due to the accurately reduced search space, especially for small datasets and biological scene. In the future, we would like to study the FPHC algorithm in terms of local learning and consider integrating multiple genomic data.

## Figures and Tables

**Table 1:** The runtime of FPNS and MMPC.

Sample Size	FPNS-D1	MMPC
50	54.2	5.17 × 10^2^
100	51.8	2.31 × 10^3^
200	52.3	3.59 × 10^3^
500	52.2	1.15 × 10^4^
1000	54.7	2.87 × 10^4^
2000	52.6	1.19 × 10^5^
5000	52.8	1.05 × 10^6^
10,000	54.2	7.58 × 10^6^
20,000	53.5	3.19 × 10^7^
50,000	53.1	2.64 × 10^8^
